# “Humanized” Stem Cell Culture Techniques: The Animal Serum Controversy

**DOI:** 10.4061/2011/504723

**Published:** 2011-04-03

**Authors:** Chandana Tekkatte, Gency Ponrose Gunasingh, K. M. Cherian, Kavitha Sankaranarayanan

**Affiliations:** ^1^Frontier Lifeline Pvt. Ltd., TICEL Biopark, Taramani, Chennai 600113, India; ^2^MIT Campus, Anna University, Chromepet, Chennai 600 044, India

## Abstract

Cellular therapy is reaching a pinnacle with an understanding of the potential of human mesenchymal stem cells (hMSCs) to regenerate damaged tissue in the body. The limited numbers of these hMSCs in currently identified sources, like bone marrow, adipose tissue, and so forth, bring forth the need for their *in vitro* culture/expansion. However, the extensive usage of supplements containing xenogeneic components in the expansion-media might pose a risk to the post-transplantation safety of patients. This warrants the necessity to identify and develop chemically defined or “humanized” supplements which would make *in vitro* cultured/processed cells relatively safer for transplantation in regenerative medicine. In this paper, we outline the various caveats associated with conventionally used supplements of xenogenic origin and also portray the possible alternatives/additives which could one day herald the dawn of a new era in the translation of *in vitro* cultured cells to therapeutic interventions.

## 1. Introduction

Human mesenchymal stem cells (hMSCs) are undoubtedly one of the most promising types of adult stem cells (ASCs) for cell-based therapies currently being tested in clinical trials for a wide range of ailments (e.g., brain and spinal cord injury, cardiovascular disease and myocardial infarction (MI), type I diabetes, multiple sclerosis, Crohn's disease, cartilage and bone injury, and graft-versus-host disease (GVHD) during bone marrow transplantation) [1]. The general practice includes isolation of hMSCs from various sources like bone marrow, adipose tissue, skeletal muscle, umbilical cord blood, umbilical cord matrix, peripheral blood, dental pulp, and amniotic fluid and its expansion under *in vitro* culture conditions. The complications in the utilization of hMSCs as therapeutic tools *in vivo*, arise due to the experimental artefacts introduced by inconsistent cell culture protocols. This necessitates the establishment of standardized culture guidelines for the isolation and expansion of hMSCs that display minimal variability in their inherent characteristic features (Table 1 [2–13]). Yet, laboratories around the world lack an internationally standardized practice for *in vitro* expansion of hMSCs, resulting in heterogeneous populations of cells and inconsistent results, both in *in vitro* studies and clinical trials.

1. With Friedenstein et al. [14] demonstrating about 4 decades ago that fibroblast-like cells grow from bone marrow when plated on FBS (Foetal Bovine Serum), animal sera remain the most ubiquitously used supplement in MSC culture medium. Despite its wide and prolonged use as an additive to chemically defined basal media for cell culture, FBS has its own economical, ethical, and scientific setbacks. This creates the necessity for the development of a better alternative to FBS, which would overcome the disadvantages.

This paper focuses on such prospective alternatives that will guarantee the safe, economical, and ethical practice of biomedical research, through the elimination of many shortcomings of FBS. Furthermore, these FBS replacements for hMSC culturing could ensure the passage of stem cell research from bench to bedside, a safe and more realistic vision. 

## 2. Scientific and Ethical Problems with Usage of Animal Serum

The usage of FBS as a medium-supplement in hMSC cell culture is widespread, despite the many disadvantages associated with it. The scientific problems encountered in cell culture due to the presence of FBS are batch-to-batch variability, fluctuating availability, unexpected cell growth characteristics, cytotoxicity of uncharacterized factors in the serum, and so forth. Another major caveat associated with the usage of animal serum is the risk of possible contamination with viruses [15], prions, bacteria, nanobacteria [16], mycoplasma, yeast, fungi, and endotoxins, some of which are impossible to remove from the serum. In addition, the presence of certain general animal-serum components (that usually enhance adhesion and spreading and occasionally inhibit cell growth) in variable amounts, such as (1) immunoglobulins, (2) transcription factors (ATF-2, SRE-ZBP, GATA-2, TFIID, Ets-1/Ets-2, E2F-1, Oct-2, p53, AP-2) [17], and (3) growth factors (platelet-derived growth factor [18, 19], insulin-like growth factors [20], epidermal growth factor [21, 22], fibroblast growth factor [23] nerve growth factor [24]), can intervene with the cellular function, growth, and the phenotypic/genotypic stability of the cultured cells [25]. Additional variability is introduced by the large number of experimental variables induced by certain identified/unidentified components in the serum [26, 27]. 

The immunogenicity of FBS-cultured cells has raised many concerns for their use in therapeutic strategies, since reports of anaphylactic or arthus-like immune reactions in patients, following the infusion of lymphocytes grown in FBS-media [28]. However, clinical trials performed on nearly 2000 patients have used FBS-cultured hMSCs, and there have been no reports on immediate signs of adverse effects or infusional toxicity [29]. Rare unfavorable occurrences and late complications however may be identified only in large groups of patients after long-time followup [30]. This may be attributed to the transfer of approximately 7–30 mg of xenogeneic proteins (from FBS) per 10^8^ hMSCs in culture [31]. Thus, thorough evaluations of long-term patient followups would give a more solid base for establishing the total safety of novel hMSC therapies from a clinical perspective. This potential risk renders the animal serum-grown cells inapt for clinical applications, proving to be a major roadblock in the passage of *in vitro* research findings to therapy. In spite of these growing concerns, FBS-cultured hMSCs have been approved by the US Food and Drug Administration (FDA) for use in human clinical trials and is widely being used in cell culture laboratories all over the globe.

Scientific difficulties aside, another major downside of FBS usage is the inhumane method of collection of blood from the foetal calves. On an average, around 1,000,000 foetal calves are killed each year for collecting around 500,000 litres of FBS [25, 32]. This is on strikingly discrepant terms with one of the major objectives of modern *in vitro *biomedical research, namely, the prevention/reduction in the usage of animals for research and testing. On conflicting terms, usage of FBS in cell culture does not help in “reducing, replacing, and refining” animal experimentation but instead asserts the exact opposite of The Three R's concept in scientific research [33]. 

The methods adopted for harvesting blood from the foetal calf are another issue of concern in the usage of FBS in animal cell culture. The most common method of blood collection is by cardiac puncture without anaesthesia, from a still-beating foetal heart. The disturbing fact is that at 70% gestation age (around 6 months of foetal development), which is most preferable for the blood collection, the calf is reported to develop awareness to pain and distress [34]. A thorough analysis by experts in the field of foetal nociception and awareness has shown that any undesirable response from the foetus can be prevented by cutting the dam's neck, thereby inducing a sudden fall in the EEG/ECoG in the dam as well as the foetus, almost simultaneously. The prevention of oxygen intake by the lungs of the calf has been reported to minimise awareness to pain [34]. Based on these studies, several measures to minimise nociception and suffering of the animals have been adopted during slaughter and foetal blood harvesting. The safe guards of blood harvesting from the foetus according to the workshop “Towards Better In Vitro Methods, The Replacement of Foetal Bovine Serum” held in April, 2003 in Utrecht, the Netherlands [35] are as follows.

- The blood collection must begin only after the EEG/ECoG of the neck-cut dam becomes flat and stays flat throughout the procedure.
- After separating the foetus from the mother, it must not be allowed to breathe air at any time during the procedure.
- The foetus must be stunned immediately if it is allowed to breathe air after being removed from the dam.

These safeguards only ensure that the calf is unconscious and desensitized to the pain caused during the cardiac puncture and blood collection. However, the number of foetal calves which are sacrificed to meet the demands is too high to be compromised. 

With cell and tissue culture becoming an indispensable tool in biomedical research, FBS-free culture techniques would benefit scientists in many aspects; by achieving a conformity of the research to Good Cell Culture Practices, it would make *in vitro *cell culture a more economical and ethical practice. 

## 3. Alternatives to Animal Serum in Stem Cell Culture

Various animal serum alternatives have been tested for their ability to sustain proliferation and differentiation of hMSCs in the recent past. The major drawback, that withholds the adaptation of FBS-free culture techniques for hMSC culture, is the incapability of hMSCs to survive in the absence of serum-specific growth factors as well as other unidentified factors in the serum. In addition, serum not only functions as a buffering agent [36] but also offers protection against certain “cytotoxic” agents by unknown mechanisms. Hence, the choice of serum for the growth of hMSCs has a profound effect on the health and quality of the cells in culture leading to a search for serum/suitable alternatives from other sources, with FBS-like properties, but not of animal origin. 

Other important qualities that are expected of an efficient medium additive are

- consistency in the concentration of the constituents,
- absence of contaminants,
- low cost,
- longer shelf-life,
- ready/easy availability.

Based on these features, the following alternatives have been proven to be potential FBS replacements that are equally good, if not better suited for hMSC culture, with each alternative having its own set of advantages and disadvantages. 

### 3.1. Human Blood-Derived Alternatives

Human-derived medium additives, that can replace FBS, have been investigated in the past couple of years and have led to the discovery of efficient alternatives such as human serum—autologous and allogeneic human serum albumin (HSA), thrombin-activated platelet releasates (t-PR), collagen-activated platelet releasates (c-PR), human platelet lysates (hPL), umbilical cord blood serum (UCBS), and autologous plasma-derived from bone marrow (APM) (Table 2 [37–47]). The major advantage of these human growth supplements is the absence of any risk of secondary effects which may be caused by FBS constituents in culture. Nevertheless, the possibility of contamination from adventitious agents in these blood-derived substituents remains a threat, but it could, however, be kept at bay by strict adherence to blood bank quality standards. However, the risk of sensitization by blood group substances or by pathogens not covered by routine blood donor testing poses another major risk in the usage of such products. Therefore, the need for implementation of various strategies to deal with these shortcomings seems inevitable. Filtration through pores of 0.2 *μ*m though would serve to eliminate certain bacteria and particulate matter would be ineffective in the removal of viruses, usually less than 0.1 *μ*m in size. Recently researchers at the Fraunhofer Institute for Mechanics of Materials IWM in Halle, Germany have developed high-precision nanoporous filter membranes of aluminium oxide with pore diameters ranging from 15 nm to 450 nm, where the removal of even the smallest viruses could be achieved [48]. Other pathogen inactivation procedures like photochemical treatment with amotosalen and ultraviolet-A light have proven effective in ensuring the highest possible quality standards [49].

High variability among human blood samples could also produce inconsistencies in hMSC growth. This could be addressed by pooling a number of individual prescreened samples, which however does not appear feasible and lacks economy of scale from a research standpoint, particularly with the rigorous quality control strategies for a large number of samples. Nonetheless, these growth supplements can function as efficacious alternatives for *in vitro *hMSC expansion in the research laboratory, till commercial ready-to-use chemical medium formulations become accessible. 

#### 3.1.1. Human Serum

Studies on hMSC isolation and expansion in 10% human autologous serum (HAS) (without any cytokines or growth factors) have proved that it is equivalent to 10% FBS in stimulating growth, making it an effective FBS alternative that is safe for therapy [9, 50, 51]. One study reported a novel method of collecting HAS in a closed bag system, thereby decreasing any risk of virus or bacterial infection and foreign protein contamination of the cultured cells [52]. Another study, conducted on the use of HAS in culture and transplant of MSCs in cellular cardiomyoplasty, has reported prevention of life-threatening arrhythmias, thereby highlighting HAS as a promising candidate for FBS replacement [53]. These evidences strongly support substitution of HAS in the place of FBS as a growth supplement for hMSCs growth and expansion. 

HAS-cultured mesenchymal stem cells remain morphologically similar to FBS-grown hMSCs and had significantly shorter population doubling times, in the range of 41–54 hours, when compared to 76–89 hours in FBS media [9]. Contradictory results were observed in the use of allogeneic serum for hMSC culture, where the cells took longer to adhere and proliferate and never reached 60% confluence [31]. This hints at the presence of some allogeneic factors in the human serum that have an inhibitory effect on hMSC growth and survival, which is not exhibited by cells grown in FBS-supplemented media despite the xenogeneic origin of FBS. 

Gene expression studies on HAS-cultured hMSCs showed diminished differentiation capability and high proliferation rates, due to the increased expression of factors like angiopoietin-like 4 gene (apoptosis inhibiting role) [9, 54]. On the other hand, cells grown in FBS media exhibit an upregulation of both the cell cycle and differentiation genes tuning them on for differentiation into osteocytes, adipocytes, and chondrocytes [9]. FBS-cultured MSCs also had several prostaglandin-synthase genes upregulated, making them favourable for use in studies that exploit the immunosuppressive properties of MSCs [9]. Nevertheless, cells cultured in HAS exhibit transcriptome stability over a long time, but a major concern regarding their use in the clinical scenario is the limited availability. A strategy that could overcome this disadvantage is the adaptation of culture conditions that are specific to the clinical requirement. For instance, in bone reconstruction, where there could be a limit to the number of progenitors required, HAS seems of interest as it favours osteoblast differentiation [55].

Pooled human AB serum has also been proven to be a feasible alternative to FBS, supporting the proliferative and differentiative ability of hMSCs in addition to retention of the MSC characteristics throughout *ex vivo* expansion culture [7]. There have been reports of successful isolation and expansion of bone marrow-derived MSCs using AB serum though growth arrest had been reported after the first passage [9, 31, 56, 57]. Adipose tissue MSCs have been efficiently grown in long-term culture with AB serum with similar cumulative population doubling as FBS [7]. These observations remain to be thoroughly evaluated from a clinical perspective before labeling pooled AB serum as an ideal FBS substitute.

There has been an interesting report on the efficacy of human AB serum/human autologous plasma in minimal quantities combined with a new serum substitute containing vegetable-derived proteins in the culture of MSCs. The growth and differentiation characteristics remained unchanged in the new combination media displaying its synergistic effects on CFU-F formation [57]. Thus, such assertions validate the need for further research into the method of using low concentrations of serum that may limit cell proliferation but still be sufficient for therapeutic applications. 

#### 3.1.2. Allogenic Umbilical Cord Blood Serum

For decades now, human umbilical cord blood has been viewed only as a source of hematopoietic stem cells, for transplantation in the treatment of various blood-related disorders and malignancies in both adult and children. The notion of using human umbilical cord blood serum (hUCBS) as a supplement for hMSC culture stemmed from the fact that cord blood is a rich source of soluble growth factors that support the growth, proliferation, and differentiation of the resident stem cell population in the foetal blood [58]. Cord blood imparts distinct characteristics to the cord blood-derived stem cells that bone marrow-derived cells do not exhibit [59], and this feature could present a unique micro environment that could support the *ex vivo* culture of hMSCs and other mammalian cell lines. In a study conducted by Bhattacharya et al. [60], it was proven that hUCBS could be used as a serum replacement for FBS in the culture of a number of mammalian cell lines. 

Human bone marrow-derived MSCs are highly proliferative cells requiring a culture medium that contains a cocktail of growth factors and proteins for their *in vitro *growth and survival. The presence of proteins like serum albumin and transferrin in high abundance is one of the reasons why hUCBS is suitable for cell culture [47]. Human serum albumin binds to several small molecules and acts as an antioxidant in most cases. The binding results in the regulation of a range of processes, such as apoptosis (negative regulation), distribution of cellular components, cellular response to starvation, chaperone binding, and transport of molecules in and out of the cells, that play vital roles in cell growth and proliferation [61]. Transferrin, on the other hand, is an iron-transporting protein that directly regulates cell growth by modulating the cell cycle at S phase [41, 62]. Higher concentration of transferrin is thus required for culture of proliferating cells than cells which are differentiating. The intake of transferrin is governed by the regulation of the transferrin receptors on the cell surface [63–65]. Apart from the proteins commonly present in the serum, 61 proteins specific in neonates have been identified in hUCBS [47]. The enhanced growth and proliferation of the hMSCs in UCBS may be due to these proteins, although their exact effect on hMSC culture is unknown. Protein precursors abundant in UCBS and their possible functions in *in vitro* MSC culture are listed in Table 3 [47, 66]. 

Cells cultured in 10% hUCBS-supplemented media have shown exponential growth with a doubling time of only 31.3 hours, compared to a longer doubling time of 44.2 hours in 10% FBS-supplemented media [10]. The enhanced growth exhibited by hMSCs in hUCBS medium, (a 32-fold increase in cell number after just 5 days of seeding (10-fold increase in 10% FBS-supplemented media)) [58], has been reported to be due to the high level of expression of cyclin D2, the cell cycle regulatory molecule which enhances the cell-cycling activities in hMSCs [10]. An increased expression of the embryonic pluripotency gene, Oct-4, in these hMSCs could be related to the high proliferation rates, in consistence with previous reports of high Oct-4 expression levels in proliferating hMSCs [67, 68]. It was also found that certain hUCBS proteins with molecular weights greater than 10 kDa had growth-enhancing effects on the hMSCs [10]. 

Cells grown in hUCBS-supplemented media retained their self-renewal capacity and sustained replicative potential, allowing nearly a 2000-fold expansion of the cells [10, 58], in contrast with FBS-cultured hMSCs that lose their colony-forming property at later passages and exhibit senescence early during *in vitro *expansion [69]. 

Electron microscopic analysis of hMSCs, grown in hUCBS, reveals some phenotypic differences at the cellular structure-level including a smaller size, a denser nuclear membrane, and meager cytoplasm as compared to FBS grown cells [10]. Nevertheless, the hUCBS-grown hMSCs hold their characteristic surface marker expression (HLA-DR, CD73, CD90, CD34, CD45, CD166, and CD105), despite their differences in structure, self-renewal, and proliferative capacities [10, 58]. 

One of the unique features of hMSCs is its multipotent nature, or, in other words, its ability to differentiate into more than one lineage. Various studies have shown that while the adipogenic differentiation potential of hMSCs grown in hUCBS medium is retained, the cells exhibit stunted adipogenesis, showing a threefold decrease in the number of oil red positive adipocytes in comparison with FBS-hMSCs [58]. On the other hand, the cells showed a more heightened osteogenic differentiation potential, owing to certain unknown stimulatory factors present in hUCBS [10]. The cells exhibited a substantial basal level of expression of osteopontin, osteocalcin, and alkaline phosphatase in the proliferative phase of growth in the hUCBS-grown hMSCs, unlike FBS-grown hMSCs where these proteins are expressed only during differentiation. Thus, hMSCs in hUCBS had a propensity to differentiate into osteocytes due to high expression of osteocalcin promoter, making them biased towards an osteogenic lineage [10]. This exceptional differentiation pattern was consistently observed in different batches of hMSCs, proving the fact that the unique stimulatory effects on the hMSCs are from certain proteins in hUCBS and not because of atypical behavior displayed by different subpopulations of hMSCs [10]. 

The variability in the differentiation patterns of hUCBS-grown hMSCs, induced by unidentified factors in hUCBS, brings about the necessity to test the efficacy of the supplement for the transdifferentiation of hMSCs into hepatocyte, cardiomyocyte, or neuronal lineages. hMSCs grown in hUCBS-supplemented media also retain the ability to differentiate into a hepatocytic lineage with an enhanced efficiency (Sankaranarayanan K. et al. (manuscript communicated and under review)). 

Apart from being an abundantly-available allogenic serum source, hUCBS has the added advantage of being free of xenogenic contaminants making the cells expanded in hUCBS-supplemented medium of therapeutic quality. The relatively easy and inexpensive isolation procedure followed to obtain clinical-grade cord serum from umbilical cord blood is one among the various advantages of using hUCBS as a medium additive. hUCBS has been extensively investigated as a safe, highly potential, and stable replacement for FBS, as it economically and ethically overcomes the shortcomings of FBS. However, the drawbacks of hUCBS are numerous: an ill-defined serum acts as an ambiguous factor; lot-to-lot variability and presence of adventitious agents that may have escaped routine screening procedures pose a threat to the purity of cells in culture. Even so, UCBS could prove to be useful as a temporary replacement for FBS until a more defined culture medium is identified for the culture of hMSCs.

#### 3.1.3. Human Platelet Derivatives

Extensive research has been conducted on human serum-free alternatives such as thrombin-activated platelet releasates (t-PR), collagen-activated platelet releasates (c-PR) and human platelet lysate (hPL). Recent studies have indicated that they could be potential FBS replacements in cell culture and could support the growth and viability of a number of different animal cell lines [70]. 

Platelets or thrombocytes are colorless, irregularly shaped nonnucleated bodies, released in blood by the fragmentation of megakaryocytes. In a healthy adult, the concentration of platelets ranges from 150,000 to 400,000 platelets per microliter. *In vivo*, these platelets play a major role in haemostasis, assisting in the formation of blood clots and blood vessels [71] and in wound healing after injury. This wound healing property of platelets is of major interest as it involves the release of multifarious growth factors like platelet-derived growth factor, transforming growth factor-*β* (TGF-*β*), fibroblast growth factor (FGF), insulin-like growth factor-1 (IGF-1), platelet-derived epidermal growth factor (EGF), vascular endothelial growth factor (VEGF), platelet factor-4 (PF-4), attachment factors (fibronectin and vitronectin), coagulation factors, mitogens, protease inhibitors, proteoglycans, and serotonin, that help in recruiting stem cells to the site of injury, thus stimulating differentiation and angiogenesis, which are critical in tissue healing [72–78]. This property of platelets, especially the presence of concentrated pools of the bioactive molecules like PDGF-*α*/*β* and TGF-*β*, is exploited for its use as a substitute for FBS in cell culture. 

Platelet lysate is generally prepared from platelet-rich plasma (PRP) isolated from uncoagulated blood (commonly used anticoagulant is Citrate). The preparation of these platelet releseates/lysates is done either by mechanical disruption or chemical lysis of the platelet membrane. The chemical lysis is carried out by the addition of Ca^2+^, collagen, or bovine thrombin to the isolated platelets thereby activating the clotting cascade. The coagulated fibrinogen is then separated from the liquid suspension, containing the platelet lysate, by centrifugation and microfiltration. The most commonly used mechanical method involves repeated freeze/thaw cycles (freezing at −20°C and thawing at <37°C), thereby disrupting the platelets through ice crystal formation. The mechanical method of platelet lysis is preferred over the chemical lysis methods due to its simplicity and cost-effectiveness. Additionally, this method eliminates the necessity for any expensive purification procedure to remove the added factors, like calcium, thrombin, or collagen, from the lysate. 

Platelet lysate also has been shown to support the growth of hybridoma cells and even enhance proliferation in a variety of tumour cell lines, transformed animal cell lines such as 3T3, SV3T3 and mouse fibroblast [79] and human endothelial cells [80]. Subcellular fractions of these platelets also have a significant positive effect on the culture of human fibroblast-like cells [81], promoting cell adhesion, proliferation, differentiation, and cell survival, thus contributing to the increase in cell adhesion, CFU-f size, and a decrease in the time taken to reach confluence [12]. It has also been reported that the usage of human platelet lysate (hPL) in hMSC culture resulted in enhanced proliferation of the cells without any alteration of their differentiation capabilities, thereby making hPL a possible candidate for *in vitro* stem cell culture for regenerative medicine [80]. 

A recent study has demonstrated that hMSCs display a more elongated, spindle-shaped morphology when cultured in hPL, thus having more number of cells per growth area [38]. The cells proliferate significantly faster in hPL media, reaching P4 in only 60 days as compared to ~100 days when cultured in FBS media [12]. This enhanced growth in hPL is attributed to differential gene expression profiles, induced by these growth factors in the hMSCs including an upregulation of cell cycle and DNA replication proteins and the down-regulation of attachment, development, differentiation and apoptotic genes [82]. 

hPL-grown MSCs also retain their multilineage potential, being still able to efficiently differentiate into osteocytes, chondrocytes, or adipocytes when subject to appropriate induction conditions [12]. However, there has been a report indicating weak differentiation of primary cultures of human muscle cells into myotubes when cultured in hPL media. This could indicate that certain unidentified factors in FBS, that are absent in hPL, may have a positive influence on primary human skeletal muscle cell culture [83]. 

An important property of hMSCs is their immunomodulatory function [38], impairing T-cell activation and, as a result, lowering the immune response after transplantation. The lack of expression of MHC II complexes on the surface of hMSCs is the reason for the lowered immune-stimulation capability of *in vitro *expanded hMSCs. It has been reported that these properties of hMSCs remain unaltered when grown in hPL [38], making them more favourable for allogeneic transplants, reducing possibilities of graft rejection and life-threatening graft-versus-host disease (GVHD). 

The use of hPL seems a first step towards a defined serum-free MSC expansion procedure; however, hPL preparations may also have donor-to-donor variations, making it difficult to standardize culture conditions. In spite of these conditions, hPL remains the most efficient FBS replacement for large-scale expansion when compared with other currently available human blood-derived alternatives [84]. In the clinical setting too, hPL could be autologously derived from the patient and be used safely for cultivating hMSCs for transplantation, reducing the patients' exposure to xenogeneic/allogenic compounds and subsequent immunological reactions. Studies on hMSC growth and differentiation capabilities using hPL have shown promising results, being safe also for reinfusion into patients [11, 84, 85]. 

### 3.2. Chemically Defined Media

Chemically defined serum-free media, supplemented with essential growth factors and nutrients to support the growth of hMSCs *in vitro*, were developed in an attempt to culture cells in the absence of animal serum. This accounts for a more controlled, consistent environment for cell growth, but few efforts were made to adapt the existing cell lines to serum-free media, resulting in the continued practice of culturing most cell lines with FBS-supplemented media. 

Unlike serum that contains a cocktail of growth factors, attachment factors, buffering agents, detoxifying agents, and compounds of varied molecular weight, a chemically-defined medium must provide these factors individually, at precise concentrations, to bring about effective proliferation without altering the cell's phenotypic characteristics. A serum alternative should sufficiently mimic the properties of the serum in culture. Essentially, all of the human-derived alternatives discussed above have the ability to both promote the adhesion of the hMSCs specifically and produce a significant enhancement in the growth rate of the cells, during *ex vivo* expansion. Thus, in the design of an ideal serum-free chemically defined medium for MSC culture, two major factors are to be considered:

1. ability of the serum-free medium to selectively promote the adhesion of MSCs and generate CFU-fs in the primary passage, completely excluding other contaminating cells from the culture dish,
2. specifically support and enhance *ex vivo* expansion of MSCs, without altering their inherent cellular characteristics or permitting the cells to enter an early replicative senescence.

Generally, chemically defined media are made using basal culture media such as DMEM *α*-MEM, onto which the required components are selectively added depending on the type of cells cultured. The selection of the additives and their concentrations, especially the growth factors, is very critical since it could variably affect the cells cultured. For example, increased level of b-FGF in the media heightens the affinity of hMSCs towards osteogenic differentiation [86]. A fine example of cytokine-influenced variations in cell growth is observed in hMSCs cultured in a serum-free medium containing FGF, PDGF, and TGF-*β*. Although TGF-*β* does not play a significant role in cell proliferation [5], only the synergistic effect of all these three growth factors results in better growth and survival of hMSCs [87]. 

Apart from growth factors, attachment and spreading factors are important for the proliferation of adherent cells in the culture. Fibronectin is a commonly used attachment factor that can be coated on the culture surface or added to the medium as a supplement. Other cost-effective and efficient methods of increasing the cell adherence include coating the surface with gelatin, alginate, or nanoscaffolds [88, 89]. Serum albumin is the most commonly added protein, which transports lipoproteins into the cell and also provides a buffering effect in the medium, followed by transferrin, an ion transporter. Hormones essential for the growth of the cells are also added in appropriate concentrations in the chemically defined media. Growth hormones, such as hydrocortisone, are added to the medium in the long-term culture of hMSCs. 

Certain minerals and other molecules which act as co-factors for numerous cellular pathways are also required as additives in chemically defined media for the *in vitro *culture of cells. These trace elements possess various enzyme binding properties and regulate specific gene expression leading to alterations in the cell division and differentiation [90]. Biotin, ethanolamine, and trace elements like selenium and iron are also routinely added in hMSC culture media. 

In general, chemically defined media for culturing adherent cell lines such as hMSCs contain albumin, growth factors, attachment factors, hormones, lipoproteins, and trace elements. A broad range of serum-free, xeno-free chemically defined media (STEMPRO MSC SFM, from Invitrogen, Carlsbad, CA; MesenCult-ACF Culture Kit (for optimal cell adherence) and MesenCult-XF Culture Kit (expansion medium) from STEMCELL Technologies, Vancouver, Canada) for hMSC culture are available commercially [39]. These commercially available media claim to be able to maintain the basic characteristics of MSCs while sustaining differentiation potentials and colony formation potentials and also exhibiting superior proliferation potentials [91]. Serum-free media containing growth factors like FGF-2, LIF, SCF, and other supplements such as pantothenate, biotin, and selenium have also been reported to support enhanced proliferation of hMSCs *in vitro* [92].

hMSCs cultured in chemically defined media display normal features even with higher growth rate, although observation of smaller cell structures and non-traditional growth pattern is common after repeated passages [5]. The desirable qualities of these chemically-defined media, apart from the ethical advantage over FBS, are their precise chemical composition, the absence of microorganisms and hard-to-remove “contaminants” like immunoglobulins, thus facilitating a controlled environment for the selective growth of cells.

Chemically defined media are designed to achieve specific effects on the cells including high proliferation rates, differentiation into specific cell types, and so forth, by varying the additives and its concentrations. A thorough analysis of the variations in the composition of these media is needed to develop a chemically defined medium for hMSC culture. The optimal media constitution mainly depends on the requirements of individual experimental conditions and may vary largely between and within cell types. This is one of the major obstacles in designing a standard chemically defined medium common for hMSC cell culture. Estimation of the exact quantities of cytokines required by the cells to sufficiently prolong the proliferative age of the hMSCs *ex vivo,* is a highly tedious process complicated by the poorly understood effects of individual growth factors on hMSC culture. This could result in changes in the inherent hMSC properties, such as HLA-DR surface antigen expression by FGF-2 and PDGF-*ββ* [93] which may in turn bring about undesired effects after transplantation. Moreover, with the compositions of the currently available synthetic media remaining as intellectual properties of the manufacturers, the prices remain relatively high (when compared to the humanized alternatives). On the other hand, these chemically defined media seem to be safer for clinical settings, in spite of the fact that not all the reagents and factors used are clinical-grade. This could best be overcome by synthesizing the cytokines as clinical-grade recombinant proteins on a large scale, while at the same time optimizing the economy of production of these therapeutic-quality reagents.

## 4. Extracellular Matrix Components

hMSCs cultured *in vitro* also express surface antigens like CD144, CD166, CD115, CD29, HLA-ABC, Sca-1, and Stro-1, other than the ones mentioned by Dominici et al. [3]. However, no available marker can be reliably used to confirm the purity of these cultured MSCs, and, hence, some experts argue that these “MSCs” represent a heterogeneous population of multipotent cells in which the real “stem-cell” component is limited [94]. Consequently, there have been numerous attempts to culture these cells on substrates that can closely mimic its *in vivo *cellular niche, which is believed to supply critical biochemical and physical signals to initiate and sustain cellular functions. Bone marrow-derived hMSCs have been reported to exhibit significantly reduced cellular aging, increased proliferation capacity, and retention of differentiation potentials when maintained *in vitro* on denatured collagen matrices [95]. The preservation of adipocytic markers and functions and the osteogenic differentiation potential of hMSCs expanded on denatured collagen type I matrix [96, 97] further underpin the significance of the matrix-mediated effects on hMSC culture. Murine MSCs have been efficiently expanded in conditions that simulate an *in vivo *bone marrow ECM microenvironment. This paper, utilizing an ECM component made from collagen types I, III, and V, syndecan-1, perlecan, fibronectin, laminin, biglycan, and decorin, emphasizes the vital role played by the bone marrow ECM in maintaining the “stemness” of MSCs [98]. 

Three-dimensional stem cell culture with synthetic hydrogel networks made of polyethylene glycol (PEG) or polyethylene glycol fumarate have also proven efficient in the maintenance of hMSCs *ex vivo,* thus demonstrating immense applications in a range of tissue engineering applications. Hydrolytically degradable PEG hydrogels, constructed via sequential step growth polymerization and photocross-linking processes, have numerous applications in “3D stem cell culture.” The PEG hydrogels remain unable to support adhesion of hMSCs, as their resistance to protein adsorption prevents the cell adhesion molecules of hMSCs from binding to the matrix. 

Adhesion and spreading of hMSCs on these synthetic PEG networks have been enhanced by incorporation of a photoreactive, phosphate-containing molecule ethylene glycol methacrylate phosphate (EGMP) [99]. Other unique molecules such as cell adhesion ligands derived from laminin and fibronectin have been tested for their individual and combined effects on hMSC spreading and viability in degradable and nondegradable PEG hydrogels, achieving promising results [100]. Similarly, the effects of FGF-2 on the viability and spreading of hMSCs cultured in 3D PEG hydrogel arrays were found to be improved when performed under varying combinations of culture parameters such as hydrogel matrix degradability, cell adhesion ligand type, and density [101]. Therefore, such alternative approaches to expanding hMSCs, guaranteeing the retention of unique characteristics, represent a novel culture technique for producing functional progenitors for various tissue engineering applications. When coupled with the serum-free or xeno-free culture conditions discussed earlier, these techniques could signify a relatively safe cellular regenerative therapeutic procedure for use in patients. 

## 5. Strategies for Culture Medium Optimization

Statistical design of experiments is a commonly used methodology to optimize *in vitro* culture conditions for a wide variety of growth systems, ranging from bacterial to animal cell culture models. Such mathematical tools for the optimization of culture media are highly warranted for, owing to the lack of customization of experimental variables to fit every designed experiment. In the absence of fine-tuning of every media constituent in a medium, used for different experimental purposes, only a very crude control of the cellular and metabolic functions involved in cell maintenance and expansion is achieved, resulting in minimal experimental success. 

To overcome the drawback of excessive time consumption of the one-variable-at-a-time method for optimization of medium components, a multivariate analysis (MVA) is followed. In MVA, one or more components are varied at a time in the cell culture medium and their resultant effects on cell growth are estimated and correlated simultaneously. Design of experiment (DoE) approach is a generally followed methodology, in which various randomized or sequential experiments are conducted to obtain statistical data for better selection of the medium components and to accurately deduce their quantitative composition in the medium. This method has proved successful for the optimization of media components [102]. 

In DoE, the first step is the selection of the components/medium factors, whose effects on the hMSCs are to be studied. These factors are either chosen randomly or in an order, based on the knowledge and experience gained from studies conducted earlier on their effects on hMSC expansion and differentiation. Such important variables, that have an impact on the growth and metabolic activities of hMSCs, can be isolated by fractional factorial design (FFD), in which only a fraction of all the possible sets of factor variables affecting the culture are considered. By this method, a large number of components can be analyzed by performing 2^*x*-1^experiments, where *x* is the number of variables, from which a subset of factors having a major effect on the hMSCs can be separated. 

After the critical components are identified by the FFD experimental methodology, the concentrations of these factors in the media are optimized. To predict the optimal concentration of these factors to be added in the media, a complicated experimental design is followed, so as to minimize the number of experiments conducted. Methods like central composite design (CCD), method of steepest ascent. are used to achieve this result. Two types of CCDs commonly utilized are central composite face-centered design (CCF) and central composite circumscribed design (CCC).

A major feature here is the interaction between these factors and their collective effect on the cells in culture. This is harder to estimate because the cells may be influenced differentially at variable concentrations of the components. Thus, to analyze the effects of these protein factors on the cells and on each other, contour plots and response surfaces are used to clearly visualize the optimum value. In this method, one or more explanatory variables are considered and their effects on the response variables (whose outcome will be affected by the explanatory variables) are analyzed by varying the composition of the explanatory variables [103]. To ensure high reproducibility of the results, the experiments are generally performed in duplicates or triplicates. This mathematical and statistical technique for optimizing culture conditions is termed the response surface methodology. 

A large number of statistical analysis tools are available that can be used to reduce the labour involved in the correlation of the results. This method has been adopted by many researchers globally to design an optimum media for cell culture. Liu et al. portray a good example of the practical application of these statistical methods for the optimization of serum-free medium for the *ex vivo* expansion and differentiation of hMSCs [104]. The study combines FFD and steepest ascent approach to successfully design a serum-free medium for the culture of umbilical cord blood-derived hMSCs. The trilineage differentiation potential of these hMSCs in the newly optimized culture medium remained unaffected thereby proving its efficacy. The optimal serum-free medium thus designed contained human serum albumin, SITE (commercially available preparation containing specific concentrations of sodium selenite, bovine insulin, human transferring, and ethanolamine), b-FGF, and Hydrocortisone in the basal IMDM medium. Though the physiological effectiveness of this serum-free medium has been proved by the growth of cord blood-derived hMSCs, it however could not support the isolation and expansion of hMSC colonies from any other source such as bone marrow, cord tissue, Wharton Jelly. This could be primarily because of the facts that

1. different physiological states of hMSCs from varied origins will warrant for different active ingredients in their culture medium,
2. extrinsic factors like inherent differences in the culture conditions and the experimental protocols conventionally followed for hMSCs from varied sources (medium composition, pH, passage intervals, etc.)

The variations in the hMSC differentiation and surface-marker expression due to differences in their *in vivo* stem cell niche [105, 106] may also affect the reproducibility of the optimum medium conditions identified. This high degree of variability in culture medium requirements for the same cell type from different sources makes the development of a generalized optimal serum-free medium for clinical-grade expansion highly challenging. Although no serious flaws have been detected in this methodology, the major obstacle in the optimization of a perfectly defined culture condition is the limited data available on the effects of all the specific growth factors on hMSCs both *in vitro *and* in vivo.* The ability to support the isolation, expansion, and differentiation of hMSCs from different sources should also be taken into account while developing a new medium, so as to obtain a generalized medium with specific components at an optimum level.

## 6. Conclusions

The therapeutic dosage of hMSCs commonly employed for infusion (e.g., in the treatment of graft-versus-host disease) is >2 × 10^6^/kg body weight of the patient [107]. Taken together with the extremely low frequency of occurrence of hMSCs in the human bone marrow (0.001 to 0.01%), the *ex-vivo *expansion of hMSCs for therapeutic applications is a must. Unfortunately, the lack of uniformity in the *in vitro *hMSC-expansion protocols presents more challenges in its ingression into the next phase in clinical research and cell-based therapy. Variables introduced during long-term *ex vivo *cell culture might have implications on the fate of hMSCs, like the heterogeneity of subpopulations of expanded cells, the disparity in expansion of the particular subsets of the populations resulting in entirely different potentials of the end products [108]. Such irregularities, where MSC properties could be modified or lost during expansion, will thus have implications on the therapeutic efficacy of hMSCs grown. 

Currently, most reported MSC-based cellular therapeutic strategies utilize hMSCs expanded in FBS-supplemented culture medium and none have reported any significant side effects due to the presence of xenogenic proteins [55, 109]. On the contrary, FBS exhibits a neuro protective function, mediated through the action of low-molecular-weight bioactive factors (like serofendic acid), which help in abolishing cytotoxic effects induced by apoptotic and necrotic signals [110]. Most regulatory agencies tolerate the presence of xenogenic components in hMSC culture media in phase I clinical trials, but later-phase studies and clinical therapies would strictly require serum-free or at least xeno-free media. Although, sequential cultivation of FBS-grown MSCs in autologous or heterologous serum can remove up to 99.99% of the xenogenic proteins [31], a residual risk still remains. 

The selection of the best replacement for FBS is crucial and depends on its ability to obtain hMSCs with characteristics similar to those of native MSCs. Consequently, this would involve identifying the progenitors in fresh bone marrow and evaluating their self-renewal and differentiation capacities with the right tools under different conditions [55, 111]. 

Some recent protocols have tried to completely shun FBS usage and shift focus towards “humanized” alternatives such as autologous serum, allogenic umbilical cord blood serum, or platelet extracts. Such changes in medium conditions might influence immunophenotypic, genotypic, and functional characteristics of hMSCs and thus warrant for extensive studies into the cell-biological aspects of the cells in different growth supplements. In addition, a thorough proteomic characterization of these humanized alternatives would aid in the identification of the specific factors that have *in vitro* growth-promoting effects on hMSCs. Consequently, the *in vitro* production of these identified proteomic factors could provide clues for the production of a defined serum-free medium for hMSC expansion. Such data derived from human supplements, along with the advancements in the statistical and computational strategies, could aid in the development of a standard, defined medium for clinical preparations of hMSCs. 

When viewed in the context of research, these human supplements could serve as cheaper alternatives for large-scale expansion of hMSCs, with better cell proliferation rates. The efficacy of these medium additives to provide clinical-grade hMSCs has also been tested for, resulting in satisfactory outcomes, thus, thrusting forward the FBS-free media bandwagon for stem cell culture. At the moment, no definitive serum-free/chemically-defined medium with all the necessary recombinant growth factors is available for hMSC amplification. Hence, these human growth supplements could serve as transitional elements in the clinical-scale production of hMSCs, until commercial, safe, off-the-shelf alternatives become available. 

The establishment of standardized protocols that conform to good cell culture practices is of high precedence in this era of Cell-based therapy and regenerative medicine. Successful transplantation of the *in vitro *expanded hMSCs and assurance of their long-term therapeutic effects, *in vivo*, is of paramount importance, to thrust this novel field forward. Once hMSC culture in the clinical setting is well-established, issues on their large-scale expansion with GMP-compliant protocols would be the next hot topic for debate. 

Therefore, the optimal culture conditions, for the efficient clinical-scale production of hMSCs that are therapeutically applicable in transplantation, immunotherapy, and regenerative medicine, still remain elusive. Despite these major questions remaining unanswered here, studying and evaluating the foundations of this issue will definitely strengthen our knowledge base, helping us make an informed choice to continue in translational research and stem cell clinical trials. Continuous research and a growing interest amongst scientists in this front would definitely fetch us a breakthrough in the ongoing pursuit for the optimal hMSC culture condition.

## Figures and Tables

**Table 1:** Typical MSC characteristics (retention/loss) in various types of Media.

MSC characteristics	Foetal bovine serum	Chemically defined media	Human serum	Umbilical cord blood serum	Human platelet lysate (HPL)/ platelet-rich plasma (PRP)
Adherence to plastic	+	+^1^	+^2^	+	+

Morphology	Spindle-shaped	More spindle-shaped	Smaller and more spindle-shaped	Elongated and more spindle- shaped^3^	Elongated and more spindle- shaped (hPL). Smaller cells in PRP

Surface antigen expression					

CD105	+	+	+	+	+
CD73	+	+	+	+	+
CD90*	+	+	+	+	+
CD45	−	−	−	−	−
CD34	−	−	−	−	−
CD14/CD11b	−	−	+/−^4^	NA	−
CD79a/CD19	−	−	NA	NA	−
HLA-DR	−	+/− ^4^	−	−	−

Tri-lineage differentiation	+	+^5^	+^6^	+^7^	+

CFU-f efficiency	Large and well spread	Varies with media components	Densely packed. Mesh-like growth pattern in later passages	Densely packed larger colonies^3^	Densely packed (hPL and PRP) Mesh-like growth pattern in later passages (PRP)

Doubling time	76–89 hrs	↓^4^	↓	↓	↓

Cost and availability	Available from certain countries, ~500 to 800 USD for 500ml.	Commercially available, expensive	Available and cheaper	Available and cheaper	Available and cheaper

References	[3, 4]	[5, 6]	[4, 7–9]	[4, 10]	[7, 11–13]

*Effect on immunosuppressive property of hMSC [2].

NA: data not available.

^1^When adhesion factors are added.

^2^Decreases in later passages.

^3^Author's observation. Unpublished data.

^4^ Affected by the factors added in the medium.

^5^Defined media for each specific differentiation are available.

^6^Differentiation of hMSCs into osteoblasts was enhanced [4].

^7^Enhanced differentiation of hMSCs into osteogenic lineage and suppressed adipogenesis also seen [10]. No data available for chondrogenic differentiation in UCBS-cultured MSCs.

**Table 2:** Comparison between the general components of serum and human platelet lysate and their effect on hMSC culture.

Components that play a major role in cell culture	Human Platelet Lysate	Human Serum*	Umbilical Cord Blood Serum*	Effects in hMSC Culture
High abundant proteins				
(i) Albumin	−	+	+	Major binding protein [40]
(ii) Transferrin	−	+	+	Iron transporter [41]
(iii) Fibronectin	−	+	+	Cell Adhesion and Migration [42]

Growth factors and cytokines				
(i) Epidermal growth factor (EGF)	+	+	+	Proliferation and differentiation
(ii) Fibroblast growth factor (FGF)	−	+^1^	+^2^	Proliferation, differentiation, and migration
(iii) Nerve growth factor (NGF)	−	+	+	Regulation of apoptosis
(iv) Vascular endothelial cell growth factor (VEGF)	+^3^	+^1^	+^2^	Proliferation and migration
(v) Platelet-derived growth factor (PDGF)	+^3^	+	+	Proliferation and migration
(vi) Insulin-like growth factors (IGFs)	+^3^	+	+	Proliferation and migration
(vii) Transforming growth factors (TGFs)	+^3^	+	+	Proliferation^4^ and cell-cell adhesion
(viii) Interleukins	+	+	+	Maintenance of stemness
(ix) Interferons	+	+	+	Differentiation and MHC antigen regulation

References	[37, 38]	[39]	[39]	[36, 37, 43–46]

^1^Released as a result of injury in the body.

^2^May be found due to its role in foetal development.

^3^Found in higher concentrations.

^4^Higher concentrations inhibit cell proliferation.

*The components of the serum have been identified but have not been characterized. Protein precursors required for maintaining foetal conditions are found only in umbilical cord blood serum [47]. The top 5 abundant proteins present in UCBS and their molecular functions are listed in Table 3.

**Table 3:** Protein precursors abundant in UCBS and their possible functions in *in vitro* MSC-culture.

S. no.	Protein precursor	*In Vitro* effects on hMSCs
(1)	Alpha-2-macroglobulin precursor	(i) Natural protease inhibitor
		(ii) Role in cell regulation and differentiation

(2)	Apolipoprotein B-100 precursor	(i) Cholesterol transporter activity,

(3)	Complement C3 precursor	(i) Positive regulation of VEGF production
		(ii) G-protein coupled receptor protein signaling pathway
		(iii) Natural protease inhibitor

(4)	Complement C5 precursor	(i) Chemotaxis, positive regulation of VEGF production
		(ii) Natural protease inhibitor
		(iii) G-protein coupled receptor protein signaling pathway
		(iv) Positive regulation of chemokine production

(5)	Isoform 1 of complement factor H precursor	(i) Negative regulation of complement C3
